# A light sheet fluorescence microscopy protocol for *Caenorhabditis elegans* larvae and adults

**DOI:** 10.3389/fcell.2022.1012820

**Published:** 2022-10-07

**Authors:** Jayson J. Smith, Isabel W. Kenny, Carsten Wolff, Rachel Cray, Abhishek Kumar, David R. Sherwood, David Q. Matus

**Affiliations:** ^1^ Department of Neurobiology, University of Chicago, Chicago, IL, United States; ^2^ University of Chicago Neuroscience Institute, Chicago, IL, United States; ^3^ Embryology: Modern Concepts and Techniques, Marine Biological Laboratory, Woods Hole, MA, United States; ^4^ Department of Biology, Duke University, Durham, NC, United States; ^5^ Marine Biological Laboratory, Woods Hole, MA, United States; ^6^ Department of Biochemistry and Cell Biology, Stony Brook University, Stony Brook, NY, United States

**Keywords:** *C. elegans*, light sheet fluorescence microscopy, BIO-133, postembryonic development, timelapse

## Abstract

Light sheet fluorescence microscopy (LSFM) has become a method of choice for live imaging because of its fast acquisition and reduced photobleaching and phototoxicity. Despite the strengths and growing availability of LSFM systems, no generalized LSFM mounting protocol has been adapted for live imaging of post-embryonic stages of *C. elegans*. A major challenge has been to develop methods to limit animal movement using a mounting media that matches the refractive index of the optical system. Here, we describe a simple mounting and immobilization protocol using a refractive-index matched UV-curable hydrogel within fluorinated ethylene propylene (FEP) tubes for efficient and reliable imaging of larval and adult *C. elegans* stages.

## Introduction

Light sheet fluorescence microscopy (LSFM) affords several advantages for live imaging of biological samples over standard epifluorescence or confocal microscopy. Whereas wide-field microscopy illuminates an entire specimen for imaging, LSFM achieves reduced phototoxicity, photobleaching, and background signal by restricting the proportion of the sample that is illuminated during acquisition. Relative to wide-field imaging, point-scanning confocal methods reduce out of focus sample illumination in the X-Y dimension by only exciting a single point in the sample at a time. To cover the whole region of interest the laser repeatedly sweeps across the sample and for each point scanned the entire Z depth is illuminated. Thus, out of focus photobleaching and phototoxicity occurs in the Z-dimension (Fischer et al., 2011). In contrast to a confocal point-scanning microscope where out of focus light is rejected by discarding unwanted emitted photons, LSFM systems generate a light sheet that selectively illuminates a narrow z-range of the sample in the desired focal plane at a given time (Fischer et al., 2011; Albert-Smet et al., 2019). This eliminates out of focus photobleaching and permits the collection of the entire fluorescence signal of a section of the sample at one time point, dramatically increasing acquisition speeds (Fischer et al., 2011). Another advantage of LSFM is the ability to acquire multi-view image data *via* multidirectional illumination, sample rotation, or a combination of both techniques (Huisken and Stainier 2009; Schmid and Huisken 2015). To overcome loss of resolution at increased tissue depths, many LSFMs are equipped with the ability to simultaneously image an individual sample from multiple sides, which can then be computationally deconvolved and reconstructed to render a single image of isotropic resolution. These technical advantages have made LSFM a popular imaging method for visualization of complex three-dimensional cells and tissues over developmental time (Keller et al., 2008; Liu et al., 2018).

Most LSFMs are equipped with two or more perpendicular illumination and detection objectives with the sample centered under or between the objectives. This unique orientation of objectives relative to the sample impedes the use of traditional flat microscopy slide mounts for the majority of LSFM systems. Samples for LSFMs are thus often embedded in a cylinder of low-melt agarose that hangs vertically between the objectives. In cases where the agarose is not dense enough to maintain its form, rigid fluorinated ethylene propylene (FEP) tubes can be used to surround the agarose cylinder to stabilize and support the agar (Kaufmann et al., 2012; Girstmair et al., 2016; Steuwe et al., 2020). The refractive indices of low-melt agarose (1.33) and FEP tubes (1.34) are well matched to the refractive index of water (1.33) and this sample mounting method works well for many organisms.

The *C. elegans* embryo has been particularly helpful in advancing the use of LSFM. For example, *C. elegans* embryogenesis was used to demonstrate the enhanced spatiotemporal resolution that is achieved using lattice light-sheet microscopy (Chen et al., 2014). Similarly, the *C. elegans* embryo facilitated showing the effectiveness of four-dimensional (4D) live imaging with the Dual Inverted Selective Plane Illumination Microscope (diSPIM) system (Kumar et al., 2014). LSFM has also advanced our understanding *C. elegans* embryogenesis (Chardès et al., 2014; Duncan et al., 2019), such as helping to reveal how the rigid egg shell contributes to asymmetrical cell divisions (Fickentscher and Weiss 2017), how circuit structures are organized within the nerve ring (the *C. elegans* brain) (Moyle et al., 2021), and how the zinc finger protein PIE-1 concentration gradient is established and maintained in the zygote (Benelli et al., 2020).

Although LSFM can also be used to capture embryogenesis in mice (Ichikawa et al., 2014; Udan et al., 2014) and zebrafish (Keller et al., 2008; Kaufmann et al., 2012; Icha et al., 2016; Pang et al., 2020), the increased tissue size and thickness, tissue pigmentation, and lack of transparency limits post-embryonic imaging in these animal models. In contrast, the small size and transparency of *C. elegans* larvae and adults makes them ideal to examine post-embryonic developmental and physiological processes. *C. elegans* is also amenable to high-resolution live imaging of genetically encoded fluorophores fused to proteins to follow protein dynamics and assess gene expression levels and patterns (Tsuyama et al., 2013; Yoshida et al., 2017; Heppert et al., 2018; Mita et al., 2019; Keeley et al., 2020). Genetically encoded fluorophores can also be conjugated to biosensors, which have been used to quantitatively monitor cell cycle state (Adikes et al., 2020) and ATP in *C. elegans* larvae (Garde et al., 2022). *C. elegans* can also be easily stained with vital dyes (Hermann et al., 2005; Schultz and Gumienny 2012; Kelley et al., 2019).

Despite the advantages of LSFM in *C. elegans* for live imaging, LSFM use in larvae and adults has been limited by the difficulty of sample mounting. Because of its ease of use and good optical properties, low melt agar has been used extensively to mount larger organisms such as zebrafish for LSFM (Kaufmann et al., 2012; Icha et al., 2016; Pang et al., 2020). More recently, it has been reported to work as a mounting media for *C. elegans* (Rieckher et al., 2018). However, small organisms such as *C. elegans* are capable of burrowing into soft agar (Burnett et al., 2018), which might be one reason that the longest time lapse reported for this method was 20 min. In addition, the 2% low-melt agarose required to immobilize worms has a gelling temperature of 24–28°C (Icha et al., 2016; Hirsinger and Steventon 2017), which extends beyond the 25°C thermal tolerance of *C. elegans* (Stiernagle 2006). To avoid high temperatures, photo-activated polyethylene glycol (PEG) hydrogels have been used to physically immobilize *C. elegans* for live imaging (Burnett et al., 2018). Yet, the refractive indices of these hydrogels are often not well-matched for the imaging media or the organism. Here we present a simple protocol for preparing and mounting post-embryonic *C. elegans* for LSFM imaging using a combination of the refractive index matched, ultraviolet (UV)-activated adhesive hydrogel BIO-133 (Han et al., 2021) and FEP tube encasement. This approach has the advantage of much longer imaging sessions (several hours) and avoids potential negative physiological effects on the worm due to heat stress. We show how this protocol can be used to time-lapse image PVD neuron dendritic branching and pruning. We also demonstrate how this protocol is applicable to imaging a variety of proteins and structures, including extracellular matrix proteins (type IV collagen and laminin), the nuclear envelope, and the distal tip cell (DTC). We expect the adoption of these methods will enable better live-imaging studies of important dynamic cell and developmental processes, such as germ stem cell biology, cell migration, cell division, and cell invasion (Sherwood and Plastino, 2018; Gordon et al., 2020; Smith et al., 2022). Furthermore, this protocol is generalizable and applicable to other organisms with little or no modifications.

## Methods

### Objectives and validation

**FIGURE 1:**
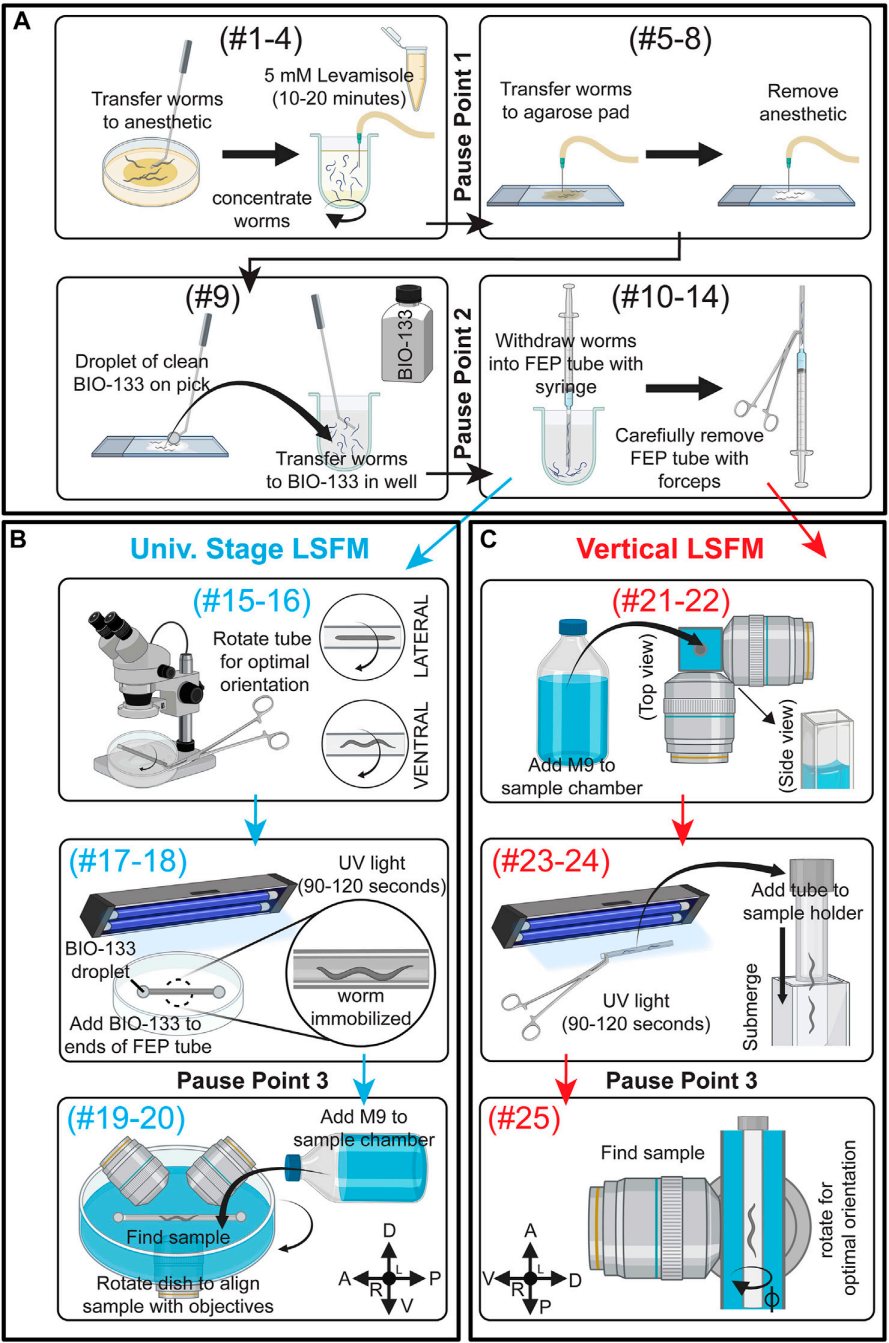
Schematic summary of *C. elegans* post-embryonic BIO-133 mounting strategies for LSFM imaging. **(A)** A schematic summary of steps #1–14 of the FEP-BIO-133 mounting protocol for time-lapse imaging of post-embryonic *C. elegans* on light sheet fluorescence microscopes, including animal anesthesia (top left, *steps #1–4*), transfer to BIO-133 (top right, *steps #5–8*), BIO-133 encapsulation (bottom left, *step #9*), and sample withdrawal into the FEP tube (bottom right, *steps #10–14*). Protocol steps #1–14 can be used for mounting samples on LSFMs configured with either a universal stage mount or a vertically-mounted sample. Pause points #1-2 in the procedure are indicated where they occur in the protocol. **(B)** A schematic summary of FEP tube-sample orientation (top, *steps #15–16*), UV-curation and bonding of FEP tube to Petri dish sample imaging chamber (middle, *steps #17–18*) and sample mounting (bottom, *Steps #19–20*) for LSFM systems equipped with a universal stage mount. After steps #1–14 **(A)**, proceed to steps #15–20. Pause point #3 is indicated. **(C)** A schematic depicting preparation for a vertically-mounted sample, including sample chamber flooding (top, *steps #21–22*), UV-curation and loading of the FEP tube into the sample holder (middle, *steps #23–24*) and rotating the FEP tube to achieve optimal sample orientation (bottom, *step #25*). After steps #1–14 **(A)**, skip steps #15–20 and proceed to steps #21–25. Pause point #3 is indicated. Created with BioRender.com.

**FIGURE 2:**
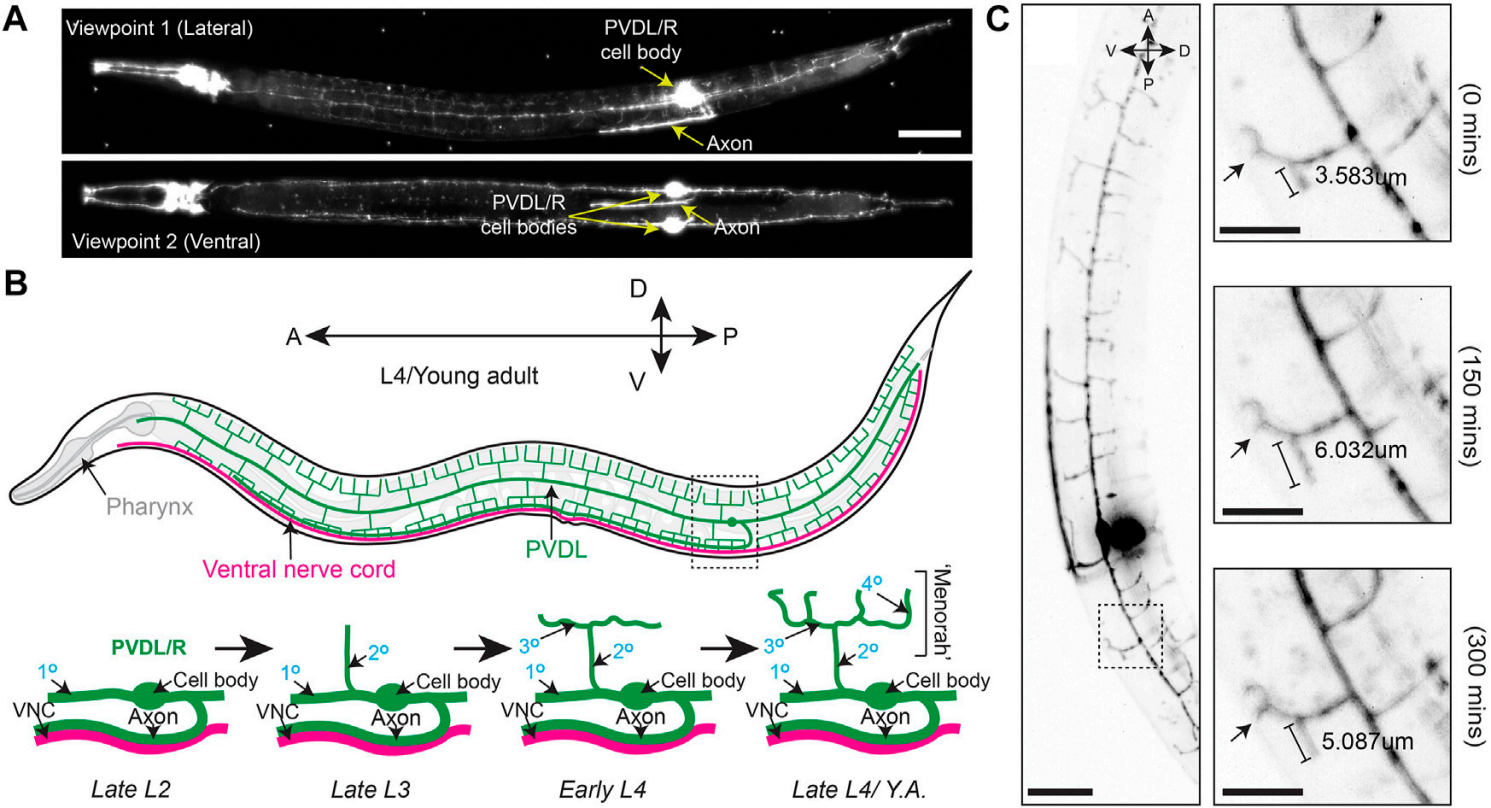
Branching and elongation of PVD neuron dendrites during a 5 h time-lapse on a DiSPIM. **(A)** LSFM Z-projections of an L4 hermaphrodite expressing *yfp::rab-10* (acquired with 40x NA 0.8 water-dipping lenses, z-step = 1 μm, 200uW/cm^2^ 488 nm laser power and 20 ms exposure) mounted using protocol Steps #1–20 on a diSPIM configured with a universal stage mount. Viewpoints were captured with imaging objectives oriented at 90° to simultaneously view the lateral and ventral aspects of the animal. Scale bar is 25 µm **(B)** (Top) A depiction of the fully elaborated PVD neurons in a young adult hermaphrodite animal. (Bottom) The developmental progression of PVD arborization focusing on the region indicated by the dashed box above. By late L2, the PVD neurons have extended their axons ventrally to contact the nerve cord and the primary (1°) dendrites have elongated along the anterior-posterior axis of the animal. The secondary (2°) dendrites branch dorsally and ventrally from the 1° dendrites by late L3. In early L4, the tertiary (3°) dendrites branch anteroposteriorly from the 2° dendrites, which is followed by the emergence of quaternary (4°) dendrites beginning in the late L4. **(C)** (Left) Timestamp from the beginning of a LSFM time-lapse in an L4 hermaphrodite expressing *yfp:rab-10* as in **(A)** (Right) Time series of 3° and 4° dendritic dynamics over the course of a 300 min LSFM timelapse (acquired with the same parameters described in A). Scale bar 25 μm, 10 µm for inset.

Our objective was to develop a procedure for immobilizing larvae and adult *C. elegans* for two-to-three-hour long LSFM time lapse imaging sessions. To accomplish this, we developed a mounting strategy that combines anesthesia, the recently developed BIO-133 UV-activated adhesive hydrogel (Han et al., 2021) and animal encasement in an FEP tube (Figure 1). This mounting method allows liquid perfusion of the worms for long term live imaging (upper limit of 3 h to avoid physiological changes that occur from starvation) and is refractive index-matched to water to minimize the light interface resulting in optimal resolution during imaging. Furthermore, this mounting protocol can be adapted to work with LSFM systems equipped with either universal stage sample mounts (Figures 1A,B) or vertical mounts (Figures 1A–C). To validate our mounting protocol, we used the diSPIM (Kumar et al., 2014) to time-lapse image the PVD neurons using a strain harboring endogenously yellow fluorescent protein (YFP) tagged RAB-10 (strain *wy1001[zf1::yfp::rab-10]*) and a membrane tethered GFP expressed in the PVD and OLL neurons (*wyIs592* [*ser-2prom3p::myr-GFP*]). *Rab-10* is a small GTPase involved in post-Golgi vesicle trafficking and is a reporter for the Golgi and early endosome vesicles in the PVD neurons (Figure 2A) (Zou et al., 2015). The multi-dendritic mechanosensory PVD neurons exist as a pair, PVDL and PVDR. Each PVD neuron sits on one side of the animal and has a single axon that extends ventrally to the nerve cord (Figure 2A, bottom). PVD dendritic branching is predictable and developmentally regulated. Specifically, early in the L2 larval stage, the PVD extends three processes—one ventrally, one anteriorly, and one posteriorly. Beginning in late L2, the anterior and posterior processes send out short extensions that will elaborate into dendritic trees that compose the non-overlapping, anteroposterior repeating structural units of the PVDs referred to as “menorahs” (Figure 2B, top) (Oren-Suissa et al., 2010). The branches of these menorah structures cover most of the body, except for the neck and head, and are labeled in the proximal-distal and chronological order in which they occur: primary (1°), secondary (2°), tertiary (3°), or quaternary (4°) (Figure 2B **, bottom**) (Smith et al., 2010). Focusing on the PVDs allowed us to validate the efficacy of this protocol with respect to anterior, midbody, and posterior immobilization as well as imaging clarity throughout LSFM-based live cell imaging. Additionally, PVD development has been the subject of previous confocal-based time lapse studies (Zou et al., 2015) and thus provided us with a point of comparison in the validation of this protocol with respect to stereotyped subcellular dynamics and structural development in a two-to-three-hour timeframe (Chen and Pan 2021; Wang et al., 2021).

We first performed time-lapse imaging of the posterior region of the PVD neuron in an L4 larval stage animal using 2-min acquisition intervals, a z-step size of 1 µm and z-range of 23 µm (Supplementary Movie S1). This allowed examination of PVD dendritic morphogenesis. We observed tertiary dendritic branch elongation (Figure 2C, bracket) as well as the growth of a quaternary branch (Figure 2C, arrow) (Smith et al., 2010; Albeg et al., 2011).

**FIGURE 3:**
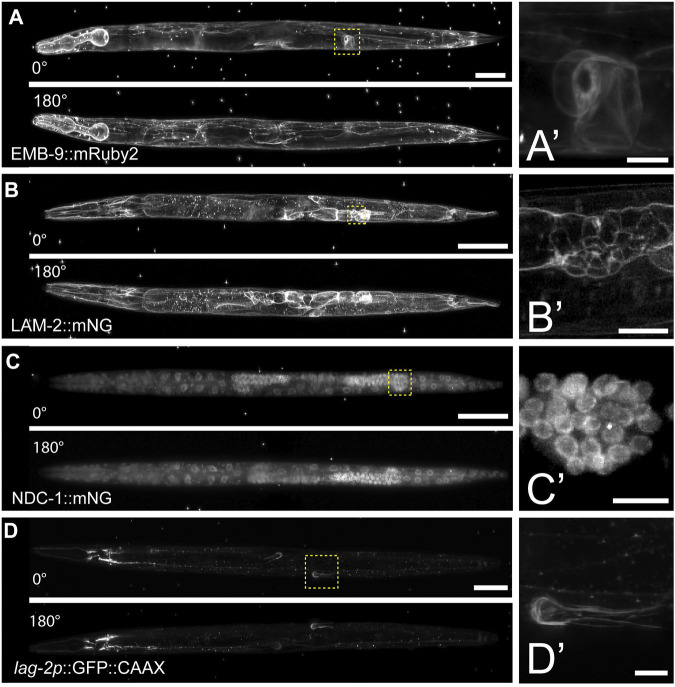
Multiview imaging of endogenously-tagged proteins in *C. elegans* young adults and larvae on a Zeiss Lightsheet 7 with a vertical mount. **(A)** Projected fluorescent images from two viewpoints on the Zeiss L7 showing endogenously-tagged type IV collagen (EMB-9:mRuby2) in a young adult hermaphrodite. The images were acquired from two angles 180° apart using a 20x NA 1.0 water dipping lens (z-step = 0.30 µm, 561 nm laser 2% power and 30 ms exposure). **(B)** Two projected images from LSFM sectioning of endogenously-tagged laminin (LAM-2:mNG) in an L4 hermaphrodite. The images were acquired from two angles 180° apart using a 20x NA 1.0 water dipping lens (z-step = 0.30 µm, 488 nm laser 1.5% power and 30 ms exposure). **(C)** Two projected images showing endogenously-tagged nucleoporin (NDC-1:mNG) in an L4 hermaphrodite. The images were acquired from two angles 180° apart using a 20x NA 1.0 water dipping lens (z-step = 0.30 µm, 488 nm laser 2% power and 50 ms exposure). **(D)** Two projected images showing distal tip cell (DTC) specific expression of membrane-tethered GFP in an adult hermaphrodite. The images were acquired from two angles 180° apart using a 20x NA 1.0 water dipping lens (z-step = 0.30 µm, 488 nm laser 7% power and 40 ms exposure). Scale bar for all images is 50 μm, 10 µm for inset. **(A′–D′)** Magnified insets of regions in the yellow dashed boxes in **(A–D)**.

To further test the compatibility of this mounting protocol with other LSFMs, we imaged multiple fluorescently tagged strains on the Zeiss Lightsheet seven from two different acquisition angles. Compared to the diSPIM, which is equipped with a universal stage, the Lightsheet 7 has a vertical tube mount, which enables sample rotation during the acquisition for multi-view imaging. Using tiling and a small step size (0.30 µm), we imaged endogenously tagged type IV collagen (EMB-9:mRuby2, Figure 3A), endogenously tagged laminin (LAM-2:mNG, Figure 3B), endogenously tagged nucleoporin (NDC-1:mNG, Figure 3C), and a cell-specific transgene expressing membrane bound GFP in the somatic distal tip cells of the germline (*lag-2p*:GFP, Figure 3D). Using a 20X, 1.0 NA objective, we observed fine morphological and cellular structures. For example, we resolved the ring of type IV collagen at the edge of the spermatheca in young adult animals (Figure 3A′’), the laminin network surrounding the epithelial cells of the L4 stage spermatheca (Figure 3B′), the distribution of nucleoporin in L4 stage germ cells (Figure 3C′), and the elaborations of the distal tip cell in the young adult stage that enwrap the germ stem cell niche (Figure 3D′). Applying Multiview-registration [Fiji plugin BigStitcher (Hörl et al., 2019)] during image processing, we were also able to create an isotropic image of type IV collagen by combining two different 180° images of the same worm (Supplementary Movie S2 **)**.

### Materials and equipment

Key resources

**Table:** 

Reagent or resource	Source	Identifier
**Bacterial strain**		
*E. coli* OP50 standard food	*Caenorhabditis* Genetics Center (CGC)	OP50
**Chemicals and Peptides**		
NaCl	Millipore Sigma	Cat #S9888
Agar A	Bio Basic	Cat # FB0010
Peptone	Gibco	Cat # 211,677
5 mg/ml cholesterol in EtOH		
KH_2_PO_4_		
NA_2_HPO_4_		
K_2_HPO_4_		
H_2_O		
MgSO_4_		
**(4)** Levamisole hydrochloride	Millipore Sigma	Cat #L9756
DIFCO™ Noble agar	VWR	Cat # 90,000–774
TetraSpeck Microspheres 0.5um	Invitrogen	Cat #T7281
**Experimental models: Strain**		
TV19023	(rab-10 (wy1001 [*zf1::yfp::rab-10*]); wyIs592 [*ser-2prom-3p::myr-GFP*]	Zou et al. (2015)
NK2585	qy152 [*emb-9::mRuby2*]	Jayadev et al. (2022)
NK2335	qy20 [*lam-2::LL::mNG*]	Keeley et al. (2020)
SBW244	sbw8 [*ndc-1::mNG*]	Mauro et al. (2021)
NK1770	qyIs353 [*lag-2p::GFP::CAAX*]; naSi2 [*mex-5p::H2B::mCherry::nos-2 3′ UTR*]	Gordon et al. (2019)
**Software and algorithms**		
Fiji Version 2.3.0	Fiji	
Imaris 9.6.0	Oxford Instruments/Bitplane	
**Microscopes and Imaging**		
Stereo microscope		
MicroManager Imaging Software	For diSPIM control and data acquisition we used the ASI diSPIM plugin within the micro-manager	https://micro-manager.org/ASIdiSPIM_Plugin: http://dispim.org/(Ardiel et al., 2017)
DiSPIM	A fiber-coupled diSPIM	http://dispim.org/(Kumar et al., 2014
DiSPIM Objective 1	40x, 0.8 NA, Water dipping	Cat # MRD07420;Nikon; Melville, NY
DiSPIM Objective 2	40x, 0.8 NA, Water dipping	Cat # MRD07420;Nikon; Melville, NY
DiSPIM Filter set	Quad band notch filter	Part # Semrock NF03-405/488/561/635E-25
ZEISS Lightsheet 7	Illumination: 10×, NA 0.2 foc (400,900–9000): Detection: Clr Plan-Apochromat 20×, 1.0 NA (421,452–9700)	Zeiss.com
**Other**		
**(13)** 15″ Aspirator Tube Assembly (for mouth pipette)	VWR^®^	Cat # 53,507–278
**(9)** Bunsen Burner		
**(3)** Eppendorf Research Plus Adjustable Vol., Single Channel Pipette (20–200 µL)	Eppendorf^®^	Cat #Z683817
**(2)** BIO-133	My Polymers Ltd	N/A
**(6)** Disposable Scalpel (for trimming FEP tubes)	Fisher Scientific	Cat #12–000-133
**(12)** Disposable glass culture tubes	VWR^®^	Cat # 47,729–572
Plastic glass culture tube caps	Port City Diagnostics	Cat #T3600CAP
**(5)** Pyrex^®^ Depression Spot Plate (85 × 100 mm)	Corning^®^	Cat # 89,090–482
**(14)** Open ended melted capillary (for mouth pipette)	KIMBLE^®^ KIMAX^®^	Cat # 34,500 99
**(15)** Kimberly-Clark Professional™ Kimtech Science™ Delicate Task Wipers	Fisher Scientific	Cat # 06–666
**(10)** Glass slides (25 × 75 × 1 mm)	Globe Scientific Inc	Cat # 1301
Heat block		
Cover glass (22 × 22 mm No. 1.5)	Fisher Brand	Cat # 12541 B
**(1)** Fluidon FEP tube (0.8/1.2 mm, 0.2 mm wall thickness)	ProLiquid, Germany	Cart # 2,001,048
**(11)** General-Purpose lab labeling tape	VWR^®^	Cat # 89097-COLOR
**(19)** Petri Dish 100 mm × 15 mm	Fisher Scientific (Falcon™)	Cat # 08–757-100D
Petri Dish 60 mm	[Worm culturing]	
**(8)** Platinum Wire (for worm pick)	SPI Supplies	Cat # 01703-AC
**(18)** UV light source (40 W)	LKE - Amazon	ASIN: B07G31SQZ7
**(16)** Specimen Forceps (serrated) [203 mm]	VWR^®^	Cat # 82,027–442
**(7)** Dissecting Stereoscope	Zeiss	Cat # Stemi 2000
**(17a)** Syringe Needle (1 in., 21 G)	BD™	Cat # 305,165
**(17b)** Syringe PP/PE (1 ml, luer slip tip)	Millipore Sigma	Cat #Z683531

M9 buffer.

**Table:** 

M9 buffer		
Reagent	Final concentration	Amount
NA_2_HPO_4_	42.2 mM	6 g
KH_2_PO_4_	22 mM	3 g
NaCl	85.5 mM	5 g
1 M MgSO_4_	1 mM	1 ml
Deionized water	-	999 ml
**Total**	**-**	**1 L**

* Autoclave to sterilize. Aliquot 50 ml into 50 ml falcon tubes. One aliquot will provide enough imaging buffer for one timelapse imaging session.

Nematode growth medium (NGM) agar plates.

**Table:** 

Nematode growth medium (NGM) agar plates		
Reagent	Final concentration	Amount
Agar A	17 g/L	34 g
Peptone	2.5 g/L	5 g
NaCl	25.66 mM	3 g
Cholesterol (5 mg/ml)	12.92 µM	2 ml
Deionized water	-	1.95 L
Total	**-**	**2 L**

*Sterilize with autoclave (60 min). Cool to 55°C in a water bath and then add 50 ml 1 M KPO_4_ buffer (pH 6.0), 2 ml 1 M MgSO_4_, and 2 ml 1 M CaCl_2_. Add 8 ml of warm NGM to each sterile plastic Petri dish using sterile technique and allow to cool. For storage, plates are inverted (NGM side up) at 4°C. NGM plates are warmed to room temperature before seeding with OP50 bacteria for feeding and culturing *C. elegans* strains.

Levamisole stock solution (anesthetic)

1. Prepare 200 mM levamisole stock solution in sterile water.
2. Aliquot 150 µL anesthetic stock solution into 1.5 ml Eppendorf tubes and store at -20°C.

4% (weight/volume) noble agar

1. Microwave 4% (weight/volume) noble agar in water to dissolve.
2. Aliquot 1 ml of the melted noble agar into disposable glass tubes and cover with foil or plastic cap. Store at room temperature for up to 3 months.
3. To use, melt noble agar in the glass tube over a Bunsen burner and add to heat block at 70°C to prevent solidification.

### Stepwise procedures

Steps 1–14 described below are shown in Figure 1A and Supplementary Video S1 *.* Video tutorials for agar pad construction, worm anesthetization, and worm transfer can also be found elsewhere (Kelley et al., 2017). All necessary materials required to perform this procedure following preparation of M9 and NGM plates are shown in Supplementary Figure S1.

**Total time:** 45–65 min

*C. elegans* stage selection and anesthesia (timing: ∼30 min)

1. Synchronize worm cultures (Porta-de-la-Riva et al., 2012) (**Time:** 15 min) or pick appropriate staged animals for imaging. (**Time:** 2–3 min)
2. Add 50 µl anesthesia solution (5 mM Levamisole in M9) to a clean well in a glass depression dish.
  Alternative to anesthesia: In addition to immobilization, the anesthetic relaxes the animals into a straight conformation, which facilitates consistent tissue geometry during imaging and permits Multiview registration. However, the use of anesthetic is not suitable for all experiments as levamisole is an acetylcholine receptor agonist that results in muscle contraction (Manjarrez and Mailler 2020). As an alternative, we found animals can be immobilized with cold temperatures by treatment at 5–7°C for ∼15 min prior BIO-133 UV-crosslinking.
3. Add 100 µl of BIO-133 to a clean well of the glass depression dish.
  Detail for precision: BIO-133 is very viscous. Use a scalpel to trim the end of a pipette tip to transfer the hydrogel more easily.
*(For Multiview registration)* In an Eppendorf tube combine 80 µl BIO-133 and 20 µl of TetraSpeck Microspheres (1:2,000 dilution), vortex thoroughly to ensure beads are evenly dispersed in BIO-133. Once mixed, add 50–100 µl of BIO-133 to a clean well.
4. Transfer 20–50 animals to the anesthesia solution and wait for 12 min or until most of the animals have ceased moving. Larvae or adults should be straight and rod-like before proceeding to the next step. (**Time**: 15–20 min)

[Pause point 1]

Transferring *C. elegans* from anesthetic to BIO-133 (Timing: ∼15 min)

5. First swirl the glass depression dish to concentrate the anesthetized animals in the center of the well and then use the mouth pipette to remove most of the liquid anesthetic from the well to further concentrate the worm bodies. (**Time:** 1–3 min).
6. Prepare an agar pad on a glass slide (See Kelley et al., 2017 for details on agar pad construction) and allow to cool for 1 min (**Time:** 1–2 min)
7. Use a mouth pipette to transfer anesthetized animals from the well in the glass depression dish to the agar pad. (**Time:** 1 min)
8. Use a mouth pipette to remove anesthetic liquid from the agar pad until animals appear nearly dry (Supplementary Figure S2). Avoid removing anesthetized animals with the anesthetic solution. (**Time:** 1–3 min)
9. Using a worm pick, gather a droplet of BIO-133 at the end of the pick. Use the BIO-133 droplet to pick and then transfer worms from the nearly dry agar pad to the well of the glass depression dish that contains the BIO-133. Carefully and vigorously swirl the worms in the BIO-133 to separate individual animals and break up any liquid droplets or bubbles that form from the worm transfer. (**Time:** 2–5 min)
  Detail for precision: Any transfer of the anesthetic or water to the BIO-133 solution will result in droplets forming in the adhesive, which will trap the animals, removing them from the hydrogel.
  Detail for precision: Transferring individual animals rather than many larvae or adults on the pick at the same time will reduce the chances of aggregation.

[Pause point 2]

Loading BIO-133-encased C. elegans into FEP tube and polymerizing the mount (Timing: ∼20 min)

10. Attach the 21-G syringe needle to the 1 ml syringe barrel.
11. Use serrated forceps to slide the FEP tube onto the 21-G syringe needle.
  Detail for precision: FEP tubes need to be rinsed and stored in double-distilled water prior to use (reference https://huiskenlab.com/sample-mounting/). Dry the outside of the tube with a Kimwipe and push air through the tube using the syringe plunger to dry the inside of the tube (**Time:** 1–3 min). Removing all water will reduce the number of droplets in the BIO-133.
  Detail for precision: Depending on the length of the FEP tube, it may be necessary to use a disposable scalpel or razor blade to trim the tube into 2–5 cm lengths. Having a shorter segment of FEP tube reduces the time required to find the sample on a LSFM system by minimizing the area containing the sample. Shorter segments of FEP tubes also bond more easily to the bottom of the plastic Petri dish that will become the imaging chamber (See steps 14–16).
12. Place the open end of the FEP tube that is attached to the syringe into the BIO-133 adhesive solution. Using the syringe plunger, draw BIO-133 into the FEP tube until the tube is ¼ full. This primes the tube and ensures that *C. elegans* larvae and adults are positioned centrally, away from the edge of the FEP tube (Step 17). (**Time:** 1–3 min)
  Detail for precision: Due to the high viscosity of the BIO-133 adhesive solution, there will be a delay between when you stop pulling the syringe plunger and when BIO-133 stops flowing into the FEP tube. If more than ¼ of the tube is filled with BIO-133 by the time the pressure is equalized, carefully expel the excess BIO-133 back into the well of the glass depression dish.
  Detail for precision: To avoid introducing air bubbles into the FEP tube, do not remove the end of the tube from the BIO-133 until you have filled the final ¾ with anesthetized animals and BIO-133 (Step 14).
13. Slowly pull the plunger to draw 5–10 anesthetized animals into the primed FEP tube. (**Time:** 2–5 min)
  Detail for precision: Due to the high viscosity of the BIO-133 adhesive solution, there will be a delay between pulling the syringe plunger and drawing anesthetized animals into the FEP tube. To avoid drawing BIO-133 and animals into the syringe barrel, stop pulling the syringe plunger when the FEP tube is ¾ full. Wait until the pressure equalizes, the FEP tube is full, and the worms stop flowing before removing the end of the FEP tube from the BIO-133 to avoid introducing air bubbles to the FEP tube.
  Detail for precision: Position the opening of the FEP tube so that the animals will be drawn into the tube longitudinally. Draw one animal up at a time and avoid overlapping animals in the tube.
  Detail for precision: Ensure that larvae and adults occupy the middle of the FEP tube since LSFM systems equipped with dip lenses will not be able to image animals that are too close to the ends of the FEP tube.
14. Remove the FEP tube from the BIO-133 and check the open end of the FEP tube and the end connected to the needle for air bubbles. The FEP tube should be filled with the adhesive solution, *C. elegans* larvae and adults, and free of air bubbles. Detach the FEP tube from the syringe with serrated forceps. (**Time:** 1–2 min)

IF USING A VERTICAL MOUNT, SKIP TO STEPS 21–25

(Steps 15–20 described below are shown in Figure 1B and Supplementary Video S2)

15. Place the FEP tube in the middle of the Petri dish. Add 2-3 drops of BIO-133 hydrogel to the FEP tube using a worm pick or pipette tip. BIO-133 will stabilize the FEP tube during and following UV-treatment. (**Time:** 1–2 min)
16. Use a stereo microscope to find the optimal orientation of the FEP tube such that your sample is as close as possible to the imaging objective. If multiple animals are mounted, roll the FEP tube in the uncured BIO-133 to achieve the orientation in which most animals are oriented properly (Figure 1B). (**Time:** 1–3 min)
17. Cure the mount with UV light for 2 min to crosslink the BIO-133 around the anesthetized animals and bond the sample-containing FEP tube to the plastic Petri dish imaging chamber. (**Time:** 2 min)

Installing the mount on an LSFM equipped with a universal stage and dipping lenses (Timing: ∼2 min)

18. After UV curing, the FEP tube should be stably attached to the surface of the plastic Petri dish and the sample should be encased in a rigid hydrogel in the FEP tube. Ensure that the FEP tube is securely attached to the Petri dish by lightly tapping it with forceps or a pipette tip. The tube should not budge or move at all before proceeding. (**Time:** 1 min)
19. Add mount to the universal stage on the LSFM system. Once the mount is resting on the universal stage, rotate the dish until your sample is optimally aligned with the imaging objectives (Figures 1B). Fasten the specimen clips to secure the Petri dish imaging chamber. (**Time:** 1 min)
20. Slowly fill the Petri dish imaging chamber with 45–50 ml room temperature M9 buffer (imaging medium), after which the dipping lens objectives can be lowered into the M9 for sample finding and subsequent imaging.

END OF PROCEDURE FOR LSFM WITH UNIVERSAL STAGE MOUNT

Installing the mount on an LSFM which requires a vertically mounted sample (Timing: ∼5 min)

(Steps 21–25 described below are shown in <b>Figure 1C</b> and step 23 (UV-curing) is shown in Supplementary Video S3)

21. Fill the LSFM media chamber with M9. (**Time:** 1 min)
  Detail for Precision: M9 can be added to the media chamber prior to starting the protocol and does not need to be replaced between samples.
22. Wipe the FEP tube containing animals in BIO-133 with a Kimwipe to remove any BIO-133 from the outside of the tube. (**Time:** 1 min)
  Detail for Precision: When possible, use forceps to handle the tube to keep the tube as clean as possible, as any smudges on the outside of the tube might impede the clarity of the imaging
23. Cure the mount with UV light for 2 min to crosslink the BIO-133 around the anesthetized animals; this can be done before or after detaching the FEP tube from the syringe needle. (**Time:** 2 min)
  Detail for Precision: Use a stereomicroscope to locate the straight, centered, and non-overlapping animals within the FEP tube. (**Time:** 1 min)
24. Attach the tube in the sample holder, keeping in mind the positions of the animals as identified in step 24. If the animals are close to the end of the tube, place the opposite end of the tube in the sample holder. (**Time:** 1 min)
25. Place the sample holder with FEP tube back into the mount so that the FEP tube is submerged in M9 and ready for sample finding and imaging. (**Time:** 1 min)

## Anticipated results

This work introduces the advantages of LSFM live imaging to long term postembryonic *C. elegans* development, including faster acquisition speed and reduced phototoxicity and photobleaching. Prior to the development of this protocol, light-sheeting imaging of *C. elegans* had been limited to embryos, very short time-lapse imaging of larvae and adults, and fixed samples (Chardès et al., 2014; Chen et al., 2014; Liu et al., 2018; Rieckher et al., 2018; Breimann et al., 2019; Duncan et al., 2019). We anticipate that adult or larval encasement in BIO-133 within an FEP tube will enable continuous LSFM imaging for at least 2 h, a time span that is comparable to that typical of confocal time lapses (Kelley et al., 2017) and which approaches the physiological limit imposed by starvation (Schindler and Sherwood 2014). Unlike the confocal time-lapse mount, this protocol exposes animals to minimal amounts (up to 2 min) of direct UV light or low temperatures (7°C for the thermal immobilization method).

In addition to the extended time of imaging and optical properties of BIO-133, other major advantages of this protocol are the low material cost, accessibility of reagents and equipment (See Materials and Equipment table), and a similarity to established low-melt agarose-based LSFM mounting protocols currently used for *C. elegans*. The mounting strategy can be easily performed with resources already present in most *C. elegans* labs, except for a UV-light source, BIO-133, and FEP tubes. Compared to a previous method which uses low-melt agarose and a glass capillary tube to immobilize larvae and adults for LSFM imaging (Rieckher et al., 2018), this mounting strategy relies on similar procedural steps but avoids exposing animals to potentially harmful temperatures and provides a submersible mount that is compatible with most LSFM configurations. A minor drawback is that there are additional steps in the BIO-133 protocol relative to the low-melt agarose-based procedure. Namely, transfer of anesthetized animals to BIO-133 requires a drying process (steps 5–9) and the mount must be UV-cured (steps 15–18 or 21–23).

Compared to the short amount of time between preparing a traditional time lapse slide and imaging a sample on a point-scanning confocal system (Kelley et al., 2017), an additional limitation of this protocol is the length of time it takes to compose and cure the mount (∼30 min) before imaging. In this protocol animals are removed from food for a longer period before imaging, which reduces the time available for timelapse before starvation by ∼30 min compared to a slide-based time-lapse mount (Kelley et al., 2017). Furthermore, since the orientation of animals within the FEP tube is fixed after UV curing, it can take multiple mounting attempts to achieve optimal animal orientation. This protocol is therefore comparatively low throughput. This is a significant drawback to the investigation of developmental processes with sensitive timing, or if there is limited time available to use an LSFM system. To shorten the time to imaging, multiple LSFM time lapse mounts can be assembled in parallel.

Another potential limitation of this protocol is that BIO-133 must be cured with light of 300–400 nm in wavelength (Han et al., 2021) Animals must thus be exposed to UV-A light, which, while less damaging to DNA than UV-B or UV-C irradiation, is known to have a photoaging effect in *C. elegans* following prolonged exposure (more than 2 h) (Prasanth et al., 2016)*.* Importantly, in this protocol we used a UV lamp which exposed the samples to UV-A light of 365 nm and 405 nm at an irradiance of approximately 6 mW/cm^2^ (Ford et al., 2021). At 2 minutes of exposure, animals received an estimated dose of UV-A light of 720 mJ/cm^2^, well-below the 15 J/cm^2^ that leads to *C. elegans* lethality (Ward et al., 2008). Although we did not monitor signs of UV-induced damage in animals treated with UV light, such as germ cell death, much higher doses of UV-A light are required to induce aging in the nematode (Prasanth et al., 2016) and all animals were viable following exposure and encapsulation in BIO-133.

Finally, we have not tested the diffusion mechanics of the activated BIO-133 hydrogel. It is possible that this protocol cannot be adapted for use in combination with diffusible cues and hormones (e.g., auxin for degron-based protein depletion) (Zhang et al., 2015; Martinez et al., 2020; Martinez and Matus 2020) or mitogens (Monsalve et al., 2019). However, pre-treatment with drugs or hormones prior to mounting animals may be sufficient to capture the desired effects, depending on the mechanics of the biological process or technique of interest. Since the ends of the FEP tubes are left open in the mount, the BIO-133 hydrogel matrix and sample should also be exposed to oxygen and media.

## Discussion

Here we describe a simple protocol for collecting high-quality post-embryonic LSFM time-lapse imaging data of larval and adult *C. elegans.* It is likely that this protocol can be adapted for the purposes of imaging other animal models, as the BIO-133 adhesive is biocompatible and FEP tubes are available in a variety of lengths and diameters. Though this method of immobilization and sample mounting provides novel opportunities for *in vivo* imaging of post-embryonic *C. elegans*, such as germ cell divisions, DTC migrations, sex myoblast migration, and anchor cell invasion, there remain a few shortcomings, such as the extended time it takes to prepare samples as discussed in the anticipated results section (Sherwood and Plastino 2018; Adikes et al., 2020; Gordon et al., 2020).

Among the many advantages to light-sheet microscopy mentioned above, this protocol enables multi-view image data *via* multidirectional illumination or sample rotation by providing access to the input image data necessary for 4D image reconstruction (Huisken and Stainier 2009; Schmid and Huisken 2015). Using 4D image reconstruction, we were able to discern the ring structure of type IV collagen in the spermathecal valve that opens to the uterus and laminin tightly covering the individual epithelial cells of the spermatheca. The BIO-133 can also be seeded with fluorescent beads (microspheres) as fiduciary markers (Preibisch et al., 2010; Wu et al., 2013) to improve multi-view image processing with greater temporal and spatial registration (Supplementary Movie S2 **)**. This protocol for *C. elegans* post-embryonic time-lapse imaging should be adaptable to any light sheet or confocal microscope that contains water dipping lenses and a universal stage mount or vertically mounted samples submerged in a sample chamber.

## Data Availability

The original contributions presented in the study are included in the article/Supplementary Material, further inquiries can be directed to the corresponding authors.

## Supplementary material

Supplementary Movie S1 Elaboration of the PVD neuron in the L4 midbody. A 5 h time-lapse of an L4 hermaphrodite expressing *yfp::rab-10.* The time-lapse was acquired on diSPIM with 40x NA 0.8 water-dipping objective lenses and images were collected every 2 min.

Supplementary Movie S2 Using microspheres for enhanced spatiotemporal resolution. An isotropic image of endogenously-tagged type IV collagen (EMB-9:mRuby2) derived from the Multiview registration of images in Figure 3A.

Supplementary Video S1
*Step 1 to step 14*.

Supplementary Video S2
*Step 15 to step 17*.

Supplementary Video S3 Step 23 (UV-curing mount for LSFMs which require vertically-mounted sample).
